# Examining the dynamics between young people’s mental health, poverty and life chances in six low- and middle-income countries: protocol for the CHANCES-6 study

**DOI:** 10.1007/s00127-021-02043-7

**Published:** 2021-07-19

**Authors:** Annette Bauer, Ricardo Araya Baltra, Mauricio Avendano Pabon, Yadira Díaz, Emily Garman, Philipp Hessel, Crick Lund, Paulo Malvasi, Alicia Matijasevich, David McDaid, A.-La Park, Cristiane Silvestre Paula, Annie Zimmerman, Sara Evans-Lacko

**Affiliations:** 1 Department of Health Policy, Care Policy and Evaluation Centre, London School of Economics and Political Science, London, UK; 2 Centre for Global Mental Health, Health Service & Population Research Department, Institute of Psychiatry, Psychology and Neuroscience, King’s College London, London, UK; 3 Department of Global Health & Social Medicine, Institute of Psychiatry, Psychology and Neuroscience, King’s College London, London, UK; 4 Department of Social and Behavioural Sciences, Harvard School of Public Health, Boston, USA; 5 Escuela de Gobierno Alberto Lleras Camargo, Universidad de Los Andes, Bogotá, Colombia; 6 Department of Psychiatry and Mental Health, Alan J Flisher Centre for Public Mental Health, University of Cape Town, Cape Town, South Africa; 7 Faculdade de Ciências Médicas da Santa Casa de São Paulo, Department of Public Health, Universidade de São Paulo, São Paulo, Brazil; 8 Faculdade de Medicina FMUSP, Departamento de Medicina Preventiva, Universidade de São Paulo, São Paulo, Brazil; 9 Programa de Pós-Graduação em Distúrbios do Desenvolvimento, Universidade Presbiteriana Mackenzie, São Paulo, Brazil

**Keywords:** Young people, Mental health, Poverty, Life chances, Cash transfer programme, Mixed-method study

## Abstract

**Purpose:**

Poverty and poor mental health are closely related and may need to be addressed together to improve the life chances of young people. There is currently little evidence about the impact of poverty-reduction interventions, such as cash transfer programmes, on improved youth mental health and life chances. The aim of the study (CHANCES-6) is to understand the impact and mechanisms of such programmes.

**Methods:**

CHANCES-6 will employ a combination of quantitative, qualitative and economic analyses. Secondary analyses of longitudinal datasets will be conducted in six low- and middle-income countries (Brazil, Colombia, Liberia, Malawi, Mexico and South Africa) to examine the impact of cash transfer programmes on mental health, and the mechanisms leading to improved life chances for young people living in poverty. Qualitative interviews and focus groups (conducted among a subset of three countries) will explore the views and experiences of young people, families and professionals with regard to poverty, mental health, life chances, and cash transfer programmes. Decision-analytic modelling will examine the potential economic case and return-on-investment from programmes. We will involve stakeholders and young people to increase the relevance of findings to national policies and practice.

**Results:**

Knowledge will be generated on the potential role of cash transfer programmes in breaking the cycle between poor mental health and poverty for young people, to improve their life chances.

**Conclusion:**

CHANCES-6 seeks to inform decisions regarding the future design and the merits of investing in poverty-reduction interventions alongside investments into the mental health of young people.

**Supplementary Information:**

The online version contains supplementary material available at 10.1007/s00127-021-02043-7.

## Background

### Mental health, poverty and life chances

Globally, one in four people are aged between 10 and 24 years, making up 1.8 billion of today’s world population [1]. Ninety percent of these young people live in low- and middle-income countries (LMICs) [2]. In LMICs, 20% live on less than $1.90 a day corresponding to about 385 million individuals [3]. Young people living in poverty face multiple forms of cumulative disadvantage—such as violence, crime, lack of educational or employment opportunities—which can significantly limit their future life chances and put them at higher risk of mental disorders [4–6]. Life chances are commonly regarded as a combination of factors that determine a young person’s opportunity to improve their quality of life [7], and have influenced current debates on child poverty and social mobility [6, 8, 9].

The nature of the relationship between poverty and mental health has been subject to academic research for decades [10]. Theories of social causation and social drift have been developed [11, 12] and—to some extent—empirically tested [13] to explain bi-directional links. The social causation theory describes how poverty leads to mental health problems by increasing exposure to violence and trauma or reducing access to social capital. Conversely, the social drift theory hypothesises that mental health problems lead to poverty because they can increase healthcare expenditure, risk of stigma, social exclusion, and loss of employment [11, 13]. Taken together, this relationship leads to a vicious cycle that makes it difficult for individuals to escape poverty and improve mental health. However, it is now widely understood that poverty is a complex multidimensional concept [14, 15] and the nature and strength of the relationship between mental health and poverty also depends on how poverty is conceptualised, measured and experienced [10].

Until now, research on poverty and mental health has focused predominantly on adult populations, and little is known about how the relationship applies to youth [16]. Yet, most mental disorders emerge during adolescence [17, 18]. Mental disorders are the leading contributor to the global disease burden for those aged 10–24 years, with self-harm and violence being the fourth [19]. Whilst mental health problems affect 10–20 per cent of children and young people worldwide [20], the burden is not equally distributed across socio-economic groups [21, 22]. It is estimated that those living in the poorest fifth of the population are twice as likely to develop mental health problems compared to those with average incomes [23].

### Interventions

Adolescence is an important time to intervene to prevent mental health problems and poverty in adulthood [24]. Because these outcomes are linked to lifetime losses in employment, other productive activities and health-related quality of life, it has been argued that even if interventions were only able to modestly reduce the incidence or severity of symptoms, their return-on-investment could be substantial [25]. Both anti-poverty programmes and mental health interventions might offer opportunities to break the cycle of poverty and mental illness [26, 27].

There is emerging evidence for promotion, prevention and treatment interventions for mental health among young people in LMICs. Interventions that promote positive mental health can be implemented successfully in school or community settings in LMICs [28]. Mental health treatments have been shown to not only reduce mental health symptoms but also help people stay in or regain employment [27, 29–31]. Overall, there is little synthesised evidence of the full range of mental health interventions for young people living in poverty, and the types of short- and long-term economic outcomes they achieve [13, 32]. However, the need for interventions to address social determinants of mental health problems, including poverty, in LMIC settings is widely recognised [33, 34]. This includes social protection measures, such as cash transfer programmes (CTPs), which have been used to target young people in some countries, and which arguably have a role in improving their mental health whilst reducing poverty [35].

CTPs provide regular direct cash payments to individuals or families identified as living in poverty. Some enforce conditions that individuals need to adhere receive the payment, such as regular health checks or school attendance. In some countries, CTPs are the main vehicle for supporting people living in poverty. During the COVID-19 pandemic, they have been used to reach out to people previously not covered or extend payments to existing beneficiaries to provide a basic safety net for those at-risk of losing their livelihoods [35]. Although evidence is only emerging, CTPs have shown that they improve mental health, for example by reducing suicide rates among adults [36–38]. Among young people, evidence suggests that they can reduce depressive symptoms and psychological distress [16, 26, 39]. Evidence of the effects of CTPs is strongly context specific with outcomes depending on population characteristics as well as programme features [27, 40–43]. For example, they can depend on the amount, regularity and duration of payments [42, 44]. Programme conditionalities, and the way they are administered can also negatively impact young people’s mental health, especially when those are difficult to achieve for the young person, and when an important proportion of family income depends on it [45]. So far, the vast majority of programmes have not intentionally planned for such effects in their design. An exception to this is the Colombian ‘Jóvenes en Acción’ (‘Youth in Action’), which offers direct monthly payments to young people for attending and completing education programmes, and incorporates mental health promotion elements such as interpersonal skills building and emotional regulation. Whilst its impact on mental health has not yet been assessed, findings from its evaluation suggest that it can effectively improve their chances of entering formal employment [46], which is a likely contributor to improved long-term mental health.

Overall, important evidence gaps remain that prevent programme funders and designers from making decisions about allocating resources so that they contribute to breaking the cycle between poverty and poor mental health for young people, and improve their long-term outcomes. In particular, there is limited knowledge in regards to the impact of CTPs on youth mental health, and the mechanisms—such as programme features, population characteristics or contextual factors—at play [27, 40–43]. Whilst there is evidence of the adverse impact of children’s mental health problems on their life chances, including those related to future (mental) health, education, skills, engagement in the labour force, social function in terms of partnership, family formation and citizenship [5], this knowledge is largely from high income countries. Furthermore, while there have been some economic evaluations of the educational impact of CTPs [47], evidence on their cost-effectiveness for health outcomes is largely absent.

Based on the summarised evidence and evidence gaps, we hypothesise that CTPs can improve youth mental health and that mental health is an important factor on the path to improved life chances. Furthermore, we hypothesise that programme features and design alter mental health and life chances outcomes, and hence influence the cost-effectiveness of programmes.

### Aims and objectives

The goal of CHANCES-6 is to advance current understanding of the dynamics between poverty, mental health and life chances in young people. We will do this by examining both the impact of poverty reduction policies on mental health, and the economic impact of mental health interventions on life chances and future risk of poverty. Findings will be used to inform decisions regarding the merits of investing in and future design of CTPs alongside investments into the mental health of young people in LMICs.

Running from September 2018 to November 2021, the project is funded by the United Kingdom’s (UK’s) Economic and Social Research Council and led by the Care Policy and Evaluation Centre at the London School of Economics and Political Science. It has partners in the UK (King’s College London) and three LMICs: Brazil (Universidade Presbiteriana Mackenzie), Colombia (Universidad de los Andes) and South Africa (University of Cape Town). Quantitative data analyses cover an additional three Latin American and African countries: Liberia, Mexico and Malawi.

CHANCES-6 seeks to address the following objectives:

- To understand the impact of CTPs, and their specific components (e.g., conditionality, age at first receipt and length of receipt) on young people’s mental health and on outcomes in early adulthood that predict life chances (objective 1);
- To understand the mechanisms and pathways from mental health and poverty to improved life chances; this includes understanding the relationship between poverty and mental health, and the extent to which improvements in mental health mediate or moderate the relationship between CTPs and life chances (objective 2);
- To investigate the economic impact of CTPs (with and without a mental health component) (objective 3);
- To understand the perspectives of professional groups involved in funding or running programmes, as well as the views and experiences of young people and their families who participate in CTPs; this includes understanding barriers in current provisions and opportunities for improving programmes (objective 4).

An overarching goal of CHANCES-6 is to inform policies and programmes. Thus, an additional study objective is:

- To engage with stakeholders and young people to ensure that the knowledge is relevant to them and can inform national policies and the design and implementation of local programmes (Objective 5).

### About the countries and their CTPs

In each of the six countries, one or several large datasets exist that are longitudinal in nature, measure the receipt of CTPs and include relevant mental health and life chances outcomes (Table 1). In Brazil, Colombia, South Africa and Mexico, CTPs refer to national programmes, whilst in Malawi and Liberia, the CTPs were introduced as part of experimental studies. Programmes in Colombia and Mexico are conditional, programmes in South Africa and Liberia unconditional and programmes in Brazil and Malawi are a combination of conditional and unconditional. An overview of the characteristics of the programmes is provided in Table 2.

**Table 1:** Description of datasets used for quantitative analysis

Dataset	Sample and youth age range for analysis	Mental health measures	Life chances measures	Cash transfer program data linkage
Itaboraí youth study—Brazil (Wave 1 + 2, 2014–2016)	*N* = 1409 youth (aged 6 to 16) Representative of Itaboraí city, Rio de Janeiro	Child behaviour checklist Strengths and difficulties questionnaire Symptoms for post-traumatic stress disorder Self-harm, suicidality	Dwelling characteristics School drop-out Extracurricular activities Exposure to violence, bullying and stressful life events Resilience Substance abuse Expectations about future	Bolsa Familia program accessed by 30% of sample
Encuesta Longitudinal de la Universidad de Los Andes—Colombia (ELCA) 2010-ongoing	*N* = 11,914 households; Youth (aged 10 to 16): *n* = 4164 nationally representative sample	EQ-5D father’s and mother’s self- reported depression or anxiety	Income, consumption Employment Educational achievement Access to financial services Substance use	Familias en Acción accessed by sample, linkage to administrative register (includes information for youth supplement)
Malawi Schooling, Income, and Health Risk Impact Evaluation Household Survey (2007–2012), 4 waves	*N* = 3810 households female youth (aged 13–22) Rural town	General Health Questionnaire-12 Mental health inventory 5	Dwelling characteristics Household assets and durables, shocks and consumption Employment Educational attainment Physical health HIV/AIDS Marriage	Randomised controlled trial with groups receiving (i) unconditional cash transfer programme, (ii) conditional cash transfer programme, (iii) nothing
South African National Income Dynamics Study (2008-ongoing), 5 waves	*N* = 28,000 households youth (aged 15–24) nationally representative	Centre for Epidemiological Studies Depression Scale (CES-D)	Employment Educational attainment Income, expenditure, assets consumption, debt, savings	Child Support Grant (aged 0–17) accessed by sample
Randomised controlled trial, Liberia (2010–2011), 2 waves	*N* = 999 men (aged 18–35); monrovia	Anti-social behaviour including aggression, impulsiveness NEO-five factor personality inventory	Income, assets, expenditure Criminal behaviour	Randomly assigned: 25% cash transfer only, 28% Cognitive Behavioural Therapy only, 25% both, 22% nothing
Progresa/oportunidades, Mexico (1997–2012) 4 waves	*N* = 6,786 housholds Youth (aged 15–17) nationally representative	Previously published depression index Subjective well-being^18^	36 parameters on micro-entrepreneurship, income, labour supply, expenditures, social status	Progresa/Oportunidades accessed by sample

**Table 2:** Description of cash transfer programmes included in CHANCES-6

	Colombia^a^	Brazil^b^	South Africa^c^	Liberia^d^	Malawi^e^	Mexico^f^
Name of cash transfer program	Familias en acción	Bolsa familia program	Child support grant	Cash transfer program provided to study participants for limited time	Zomba cash transfer programme	Progresa/oportunidades
Population (families, young people)	Families	Families	Children	Young men	Girls and young women	Families
Objectives	To overcome poverty and strengthen human capital	To promote social inclusion and strengthen human capital	To ensure basic needs of children < 18 years are met (as part of broader poverty reduction strategy)	To stimulate legal self-employment	To increase schooling and health of female adolescents and young adults	To improve child nutrition, health and education
Households/individuals reached (estimate)	2.7 million families	11 million households, 46 million people	12 million children	Experimental: *N* = 999 male offenders aged 18–35	Experimental: *N* = 3796 female adolescents and young adults	5.8 million households
Coverage	17.5% of total population	20% of total population	78% of eligible children	Not applicable	Not applicable	20% of total population
Budget of programme as proportion of GDP	0.19%	0.5%	7.5%	Not applicable	Not applicable	0.5%
Benefits	USD 17 to USD 33 per month	USD 20 per month/person plus USD 10 per child and 15 per young person aged 16–17 (for conditional program); average USD 50 per family	USD 28 per month	USD 100 per month (two one off payments in 2 consecutive months)	USD 4 to 10 for parent; USD 1 to 5 for adolescent/ young adult; plus school fees	USD 10.5 to USD 66 per month
Recipient	Caregiver of child or young person	Caregiver of child or young person	Caregiver of child	Young person (male)	Caregivers, young person (female)	Female head of household
Eligibility	Families in poverty, displaced by internal conflict and/or from indigenous communities with members under 18 years old	Poor families: monthly per capita income < 40 USD (eligible for conditional part of programme) or < 20 USD (eligible for unconditional part of programme)	Child < 18 years; caregiver’s yearly income < USD 3,275 (single) or < USD 6,555 (combined with spouse)	High risk (defined by their involvement in drug use and dealing and other types of offences)	Age 13–22, never married, enrolled in primary/secondary school or recent dropout	Poor families with child < 18 years
Identification methods	Geographical; identification system (SISBEN)	Geographical; means test income threshold	Proxy means test	Not applicable	Not applicable	Geographical; proxy means test (questionnaire ENCASEH)
Conditional or unconditional	Conditional	Mix: unconditional for extremely poor; conditional for poor families	Unconditional	Unconditional	Mix: conditional and unconditional arms in study	Conditional
Conditionalities	Child health checks; regular school attendance (80%)	Regular medical consultation, vaccinations, school attendance (75–85%)	Not applicable	Not applicable	Conditional arm Regular school attendance (80%)	Regular school attendance (85%); regular medical check ups
Monitoring	Information systems	Nutritional surveillance; vaccination monitoring	Not applicable	Not applicable	Self-reported; school attendance records	Compliance checks (attendance cards)

^a^Fiszbein A and Schady N (2009)[55]

^b^Ministério da Cidadania (2019), Soares S (2012) [56, 57]

^c^NIDS (2019), Seekings (2007) [58, 59]; South African Government website: https://www.gov.za/services/child-care-social-benefits/child-support-grant

^d^Blattman et al. (2016) [60]

^e^Baird S et al. (2011), Angeles et al. (2019) [26, 45]

^f^Fiszbein A and Schady N (2009) [55]

With regards to the country context, in which programmes operate, population size varies substantially from 5 million (Liberia) to 205 million (Brazil), whilst the proportion of young people is relatively similar (16–22%) across countries. In terms of health risks for young people (15–24 years), leading factors include HIV (South Africa), binge drinking (Brazil), and child marriage and teenage pregnancies (Malawi and Liberia). Brazil ranks highest with regards to estimated burden of mental disorders (measured in disability adjusted life years) as well as investments into mental health service infrastructure. Table 3 presents an overview of the countries’ data for important mental health, poverty and life chances indicators.

**Table 3:** Overview of population, poverty, mental health and life chances indicators for CHANCES-6 countries

	Brazil	Colombia	South Africa	Liberia	Malawi	Mexico
Population^g^						
Total population	205,962,108	48,228,697	55,291,225	4,499,621	18,143,315	125,890,949
Population 15–24 years	33,689,000	8,711,000	9,820,000	983,000	3,886,000	22,139,000
Proportion 15–24 years, in %	18	16	18	22	21	18
Proportion (all ages) living in rural areas, in %	15	23	35	50	84	21
Poverty and income inequality^h^						
GDP per capita (2018), in USD	8,921	6,651	6,374	674	389	9,698
Poverty headcount ratio at USD 1.90 a day (2011 PPP), in % of population	3.4	4.5	18.9	38.6	71.7	3.8
GINI Index (2017)	53.3	49.7	63	35.3	44.7	48.3
Mental health (MH)^i^						
MH expenditure per person, in USD	1.4	Not reported	6.7 (12.4^j^)	0.02	Not reported	Not reported
Government’s expenditure on MH as proportion of total government health expenditure, in %	1	Not reported	3 (5^k^)	2.4	Not reported	Not reported
Burden of mental disorders (DALYs); per 100,000	3,593	3,526	3,191	2,298	Not reported	2,368
Plan or strategy for child and/or adolescent mental health	Yes^l^	Yes	Yes	Yes	Not reported	No
Suicide mortality rate; per 100,000	6.5	7.2	11.6	6.8	Not reported	5.1
Psychiatrists per 100,000	3.16	1.84	1.52 (0.31 among uninsured population^m^)	0.04	0.01	0.21
Child psychiatrists per 100,000	(38^n^)	Not reported	0.08 (0.02 among uninsured population^o^)	Not reported	Not reported	0.03
Psychologists per 100,000	12.37	Not reported	Not reported (0.97 among uninsured population^p^)	Not reported	0.02	3.46
Other paid MH workers per 100,000	243	Not reported	Not reported	4.56	Not reported	0.25
Total number of mental health professionals	653,329	885	Not reported	560	Not reported	5,541
Total number of mental health workers per 100,000	318	1.84	Not reported	12.45	Not reported	4.4
Outpatient facilities for children and adolescents (total)	223	Not reported	Not reported	6	Not reported	26
Life chances^q^						
Not in education, employment or training (NEET), 15–14 years old, in %	24	23	32	Not reported	Not reported	18
Youth unemployment, in %	29	19	53	3	7	7
Labour force participation (ages 15–24) in %	55	52	26	30	64	44
Employment to population ratio, ages 15–24 total, in %	39.5	42.9	11.9	55.6	27.4	40.7
HIV prevalence (ages 15–24), in %	Female: 15–19 years 3.2, 20–24 years 10.3 Male: 15–19 years 7.0 20–24 years 36.2^r^	Female: 0.1 Male: 0.1	Female: 11.3 Male: 3.7	Female: 0.8 Male: 0.4	Female: 4.3 Male: 2	Female: 0.1 Male: 0.1
Prevalence binge alcohol use (ages 15–19), in %	Female: 32.3 Male: 32.7	Female: 20 Male: 29	Female: 11.2 Male: 15.8	Female: 13.4 Male: 17.8	Female: 5.0 Male: 16.3	Female: 11.8 Male: 21.6
Adolescent life births per 1000, 15–19 years	66.8	41.6	40.4	104.8	142.7	60.5
Females (ages 20–24) in marriage before age 18 years, in %	26^s^	23.4	Not reported	35.9	42.1	26.1

^g^World Bank Group [61]

^h^Ibid

^i^World Health Organisation [62]

^j^In brackets a more recent figure is provided from Docrat et al. 2019 [63]. This figure is shown brackets because it is not from the same year or source as the figures for the other countries, which relate to 2017.

^k^Ibid

^l^Whilst the WHO source states that no such plan is in place, the following documents have been produced by the Brazilian Ministry of Health (2011, 2014) http://bvsms.saude.gov.br/bvs/saudelegis/gm/2011/prt3088_23_12_2011_rep.html

http://bvsms.saude.gov.br/bvs/publicacoes/atencao_psicossocial_criancas_adolescentes_sus.pdf

^m^Docrat et al. 2019 [63]

^n^Demografia Médica no Brasil 2018. São Paulo, SP: FMUSP, CFM, Cremesp, 2018. 286 p. ISBN: 978–85-87,077–55-4; available from http://www.epsjv.fiocruz.br/sites/default/files/files/DemografiaMedica2018%20(3).pdf (last accessed 6 April 2020).

^o^Ibid

^p^Ibid

^q^World Bank Open Data [1]; Azzopardi et al. 2019 [2]

^r^Ministério de Saúde Brasil (2018), Boletim epidemiológico HIV/Aids 2018, http://www.aids.gov.br/pt-br/pub/2018/boletim-epidemiologico-hivaids-2018;

^s^Plan International (2019), Tirando o véu Estudo sobre casamento infantile no brasil https://plan.org.br/wp-content/uploads/2019/06/Estudo-Casamento-Infantil-Brasil_final.pdf

## Methods

### General approach

**Fig. 1:**
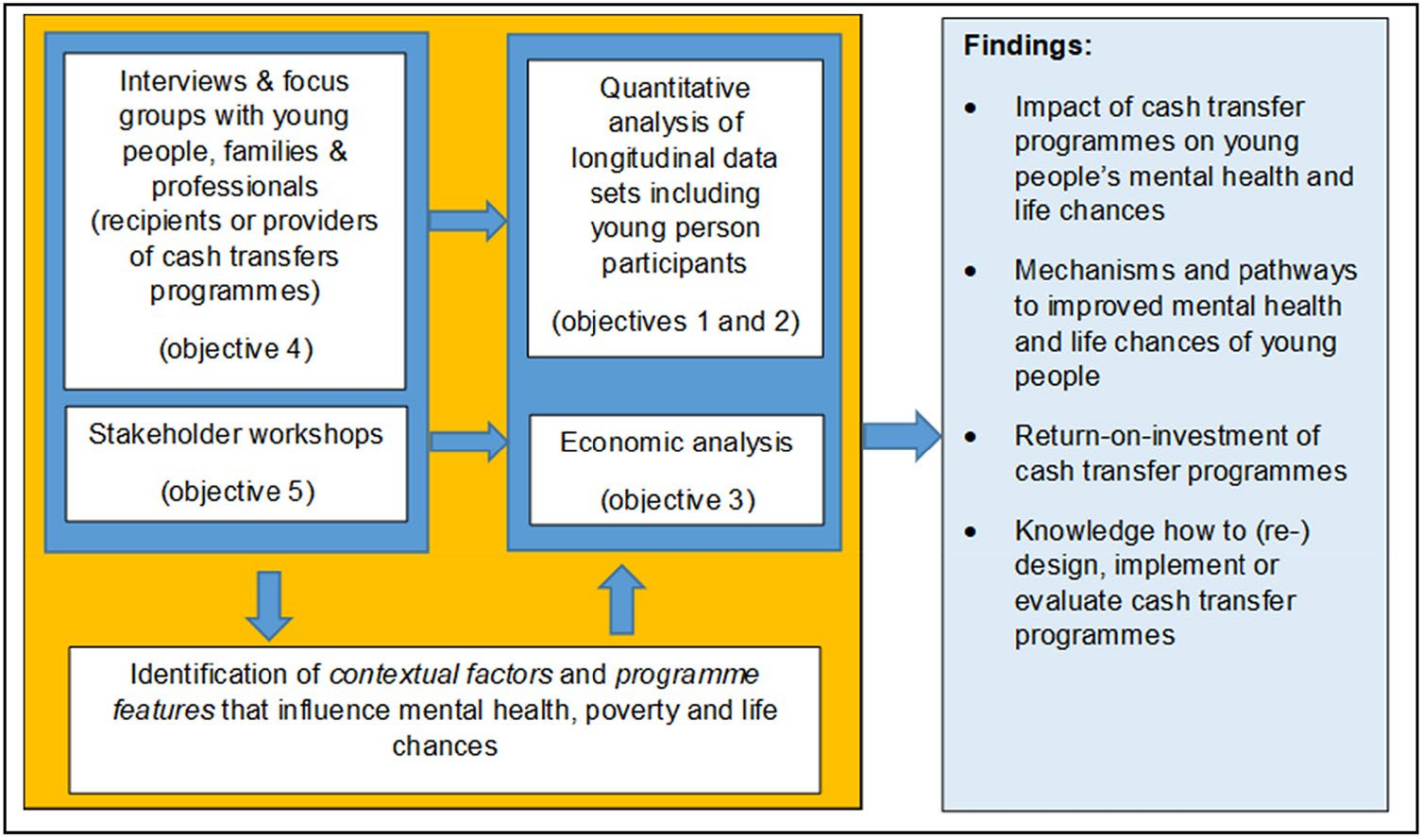
Overview of the general approach

Our approach to addressing the five objectives involves a range of methods and work streams. The first two objectives will be addressed primarily through quantitative (statistical) analyses of data on recipients of CTPs. Important concepts emerging from the qualitative analysis, i.e., interviews and focus groups with providers and recipients of CTPs (objective 4), as well as from stakeholder consultations (objective 5) and the scientific literature will inform the interpretation of findings from quantitative analyses. This includes knowledge about contextual factors and programme features likely to explain differences in findings on impacts. Findings from the qualitative analysis will inform interpretation of results from the quantitative analysis. The economic analysis conducted (objective 3) will be informed by both the quantitative and qualitative analyses, as well as stakeholder consultation. Figure 1 provides an overview of the approach. This study will follow a triangulation design [48], in which quantitative and qualitative parts are conducted mainly in parallel and we use and analyse multiple sources of data together to more comprehensively address our research question and to increase validity of findings. The main interaction and integration between the quantitative and qualitative methods will take place in the analysis and interpretation of the quantitative findings. However, there are additional interaction points as triangulation follows a flexible approach that allows findings to emerge at different stages and from the different parts of the research to address interconnected questions of the role of contextual factors, mechanisms and impacts.

### Investigating the impact of CTPs (Objective 1)

In quantitative (statistical) analyses, we will explore effects of the CTPs on mental health and on life chances outcomes by first examining impacts overall and then by mental health status, adjusting for all relevant covariates. We will first conduct country-specific analysis on each CTP, and then conduct analysis based on data that are harmonised across all countries. The primary data sources for the quantitative analysis will be data from intervention and population panel studies in each of the six countries (Table 1). Table 4 presents the socio-demographic characteristics of young people and their households from the population panel studies in Brazilian, Colombian and South African, including a comparison of characteristics of young people with and without mental health problems.

**Table 4:** Socio-demographic characteristics of young people and their households for Brazil, Colombia, South Africa; comparison of young people with and without mental health problems

	Brazil^a^						Colombia^b^						South Africa^c^						
	Total (*N* = 1189)		Mental health problems^d^ (*N* = 155)		No mental health problems (*N* = 1034)		Total (*N* = 6430)		Mental health problems^d^ (*N* = 642)		No mental health problems (*N* = 5788)		Total (*N* = 2452)		Mental health problems^d^ (*N* = 394)			No mental health problems (*N* = 2058)	
	*N*	%	*N*	%	*N*	%	*N*	%	*N*	%	*N*	%	*N*	%	*N*	%		*N*	%
Single mother	421	35	57	37	364	35	–	–	–	–	–	–	–	–	–	–		–	–
Socio-economic group^e^																			
Low	662	58	93	60	569	55	1136	18	302	47	2877	50	–	–	–	–		–	–
Middle	458	39	54	35	404	39	1663	26	333	52	2818	49	–	–	–	–		–	–
High	69	6	8	5	61	6	3534	56	7	1	93	2	–	–	–	–		–	–
Household income under poverty line	–	–	–	–	–	–	–	–	–	–	–	–	1169	42	211	51		958	41
Mother’s education																			
No/basic	464	39	65	42	399	39	–	–	–	–	–	–	–	–	–	–		–	–
Middle	354	30	42	27	312	30	–	–	–	–	–	–	–	–	–	–		–	–
High	371	31	48	31	323	31	–	–	–	–	–	–	–	–	–	–		–	–
Father’s or head of household education																			
No/basic	511	43	73	47	438	43	–	–	–	–	–	–	–	–	–	–		–	–
Middle	372	31	38	25	334	32	–	–	–	–	–	–	–	–	–	–		–	–
High	303	26	44	28	259	25	–	–	–	–	–	–	–	–	–	–		–	–
Mother unemployment (paid work in the past 30 days)	484	41	70	45	414	40	229	4	24	4	205	4	–	–	–	–		–	–
Father unemployment (paid work in the past 30 days)	98	13	19	19	79	12	460	7	57	9	403	7	–	–	–	–		–	–
Ethnic group																			
Black African	–	–	–	–	–	–	–	–	–	–	–	–	2091	84	348	91		1743	83
‘Coloured’^f^	–	–	–	–	–	–	–	–	–	–	–	–	310	8	41	6		269	9
Indian	–	–	–	–	–	–	–	–	–	–	–	–	19	2	0	0		19	2
White	–	–	–	–	–	–	–	–	–	–	–	–	32	5	5	3		27	6
Resides in rural area	–	–	–	–	–	–	–	–	–	–	–	–	1446	46	246	53		1200	48
No health insurance	–	–	–	–	–	–	–	–	–	–	–	–	2278	88	378	93		1900	87
Overcrowding																			
Average number of persons in one room	1.1	57	1.3	77	1.0	52	–	–	–	–	–	–	1.7	–	2.1	–		1.7	–
Number of people in one room > 2	–	–	–	–	–	–	1466	23	153	24	1293	22	–	–	–	–		–	–
Quality of housing and access to house																			
Street to house not paved or asphalted	801	67	115	74	40	66	–	–	–	–	–	–	–	–	–	–		–	–
Inadequate external walls^g^	–	–	–	–	–	–	–	–	–	–	–	–	565	19	90	22		475	19
Access to water and sanitary facilities, public utilities																			
No piped/running water access	820	69	106	68	714	69	1336	21	113	18	1223	21	–	–	–	–		–	–
Without access to sanitary service	–	–	–	–	–	–	842	13	113	18	827	14	386	12	76	15		310	11
Unimproved water system^g^	–	–	–	–	–	–	–	–	–	–	–	–	870	30	139	34		731	30
Unimproved sewage system^g^	–	–	–	–	–	–	–	–	–	–	–	–							
Child education																			
School attendance	1174	99	151	97	1034	99	–	–	–	–	–	–	–	–	–	–		–	–
School drop–out	10	1	3	2	7	1	–	–	–	–	–	–	–	–	-	–		–	–
School repetition	151	13	28	19	123	12	–	–	–	–	–	–	–	–	–	–		–	–
Education (in years)	–	–	–	–	–	–	–	–	–	–	–	–	9	–	8.9	–		9	–
≥ 2 years behind expected grade	–	–	–	–	–	–	–	–	–	–	–	–	1315	49	210		53	1105	54
Not in education, training or employment	–	–	–	–	–	–	–	–	–	–	–	–	356	13	68	18		288	12

^a^Itaboraí Youth Study-Brazil: Comparison of socioeconomic characteristics among young people with and without mental health problems (2015); socioeconomic characteristics were only assessed at the second wave of the study

^b^Encuesta Longitudinal de la Universidad de Los Andes—Colombia (ELCA) 2016. Sample age 11–16

^c^South African National Income Dynamics Study (2008-ongoing), 5 waves Sample at Wave 3, age 15–19 (all populations). Estimates (percentages and standard deviations) are weighted using sampling weights from Wave 3

^d^Brazil and Colombia: Refers to standard cut-off scores of the Strengths and Difficulties Questionnaire (SDQ); young people without mental health problems include those scoring in the normal and borderline range; South Africa: Refers to the Centre for Epidemiological Studies Depression Scale (CES-D) and a cut off score of 12

^e^For Colombia, socio-economic strata represents the official socio-economic classification used by the government. The Encuesta Longitudinal Colombiana (ELCA) data only includes households from the first 4 (out of 6) socio-economic strata

^f^In South Africa, the term ‘coloured’ is used to identify a group of people with mixed black and white ethnicity who have a relatively distinct cultural identity, particularly in the Western Cape. In the apartheid era, these racial categories were used by the government to legitimise state-sponsored oppression of ‘black’ and ‘coloured’ people. As the effect of these practices on health and access to resources may still be apparent, these categories are used within the context of the study

^g^Inadequate external walls refers to: exterior walls built of untreated wood, boards, planks, vegetation (e.g., guadua), zinc, cloth, cardboard, waste material or no exterior walls (urban household); exterior walls built of vegetation (e.g., guadua) zinc, cloth, cardboard, waste materials or no exterior walls (rural household). Unimproved water system refers to: no connection to public water systems; rural households: water used for the preparation of food obtained from wells, rainwater, spring sources, water tanks, water carriers or other sources. Unimproved sewage system refers to: no public sewer system; rural households: toilet without a sewer connection, latrine or no sewage system

All studies have used validated measures of poverty, mental health and life chances variables from well-established datasets. Based on these secondary datasets, we will investigate the impact of CTPs on mental health, and on life chances variables for young people. Different age ranges will be considered depending on eligibility criteria for the CTP and study design (Table 1). As much as this is possible mental health variables that indicate conditions will be analysed on a continuum “from mild, time-limited distress to chronic, progressive and severely disabling conditions” [18]. In addition to assessing mental health conditions, variables which focus on related positive aspects of mental health such as life satisfaction, self-esteem, self-efficacy, and resilience will be analysed. With regard to life chances, a range of variables are measured in studies that provide indicators of the present situation and future of young person participants. These include educational achievements, employment status, income, substance misuse, living arrangements and relationship status (Table 1). In terms of poverty, relevant variables exist in each of the datasets, which allows us to adopt a multi-dimensional approach to poverty.

Our data analysis strategies will be based on quasi-experimental evaluation techniques [49] and other epidemiological approaches. As examples of quasi-experimental techniques we will identify so-called ‘discontinuities’ in the eligibility criteria for CTPs (often based, in parts, on an income or poverty threshold) and use a regression discontinuity design [8]. For some programmes, we will use a difference-in-differences design, incorporating propensity score matching when appropriate. As for other epidemiological approaches, we will use different ways of estimating associations and causal effects of programs with cross-sectional and longitudinal data. The analytic method will be selected based on: (a) presence (or not) of necessary information/variables as well as number of observations for applying each quasi-experimental method and (b) verification of main identification assumptions underlying each method. Where the data meet the criteria of more than one method, we will compare results of all available methods.

Data harmonisation will include identifying commonalities and differences in mental health, life chances and poverty measures, and harmonising them across datasets, for example, using standardised percentile scores. Since datasets refer to different time periods, collected at different waves, and covering different age ranges, a selection will be made for the harmonised dataset, choosing data for time periods and age ranges most closely aligned with each other. The harmonisation process will involve collating shared variables (regarding the CTPs, mental health, life chances, poverty and socioeconomic status) and using the differences between programmes to answer key questions about their effect on mental health. This will allow comparisons across cultures and across CTPs (e.g., conditionality, age at receipt, length of receipt) to determine which features of the CTP are associated with better improvements in mental health and life chances. Online resource 2 provides an example of how data might be harmonised, by demonstrating this for selected datasets and indicators.

### Delineating pathways and identifying mechanisms (Objective 2)

We will conceptualise and assess pathways that explain relationships between poverty, mental health and future life chances, and the influence CTPs have on them. This will be done primarily using quantitative analyses of datasets mentioned above (Table 1). As with the analysis of impact, parameters will be informed by qualitative findings, as well as scientific literature and stakeholder consultation. In addition, qualitative data could provide a broader conceptualisation of pathways and mechanisms, including those that cannot be tested quantitatively through our analyses (but that might inform future data collection and analyses).

First, we will carry out a systematic review of the literature on the impact of CTPs on mental health outcomes of young people, focusing on LMICs. We will then develop a conceptual framework of hypothesised pathways and mechanisms based on those contextual factors, conditionalities and features of CTPs identified as important in previous evaluations of CTPs and mental health programmes, other relevant scientific literature and views and experiences from young people and professionals involved in or knowledgeable of CTPs.

Overlaps between data gathered from the reviewed literature and our own knowledge (including knowledge based on data collected by that time) will be used to confirm the importance of pathways. Where data from different sources diverge, this will be also highlighted.

Next, where possible, we will test some of the mechanisms linking mental health and poverty to improved life chances. Potential variables to consider as having a role in those pathways include:

- Contextual factors: e.g., unemployment, social cohesion, family functioning;
- Conditionalities: e.g., school attendance and child health visits; and
- Other programme features: e.g., amount of money; length of time receiving the cash transfer; ways of monitoring compliance

We will develop statistical models using recommended methods for mediation and moderation [50]. For example, we will examine whether CTPs are associated with improvements in mental health and if these improvements mediate any improvement in future life chances.

### Simulating cost-effectiveness of CTPs and mental health interventions (Objective 3)

Decision analytical modelling will be conducted to estimate the return-on-investment to the public purse from investing in country specific CTPs given any potential association we identify with mental health outcomes and life chances. This will be compared with expected outcomes and public purse costs associated with no intervention. In addition, the return-on-investment from investing in CTPs will be compared with alternative or complementary investment in selected effective mental health interventions in LMIC contexts. Such interventions will be identified in published systematic reviews and meta-analyses.

Effect sizes identified in the statistical analysis in Objective 2 will be combined with longitudinal trajectories of mental health and life chances identified in each of the longitudinal data sources (Table 1) to estimate potential long-term outcomes. Long-term monetary values will be attached to different life chances outcomes where possible. An example would be to estimate the value of higher rates of school completion for wealth accumulation and income through to adulthood. We will draw on published literature, e.g., costs reported in previous economic analyses of CTPs [47], as well as statistical reports relating to CTPs in the six countries, to estimate their administrative costs. The specific time frame for the modelling will depend on data availability. Costs and outcomes beyond 1 year will be discounted and all monetary values will be reported in purchasing power parity adjusted international dollars.

Modelling will also take account of implementation and scale-up costs in line with previous work [51]. We will also vary underlying assumptions using both deterministic and probabilistic sensitivity analysis to reflect uncertainty on both effectiveness estimates and cost distributions. To increase policy relevance, and after engagement with stakeholders (Objective 5) we will also model specific conservative and optimistic scenarios. This could include varying assumptions on uptake rates to reflect themes in qualitative analysis with young people, families and professionals set out in Objective 4.

### Understanding young people’s, families’ and professionals’ experiences (Objective 4)

We will conduct semi-structured interviews and focus groups with young people, families and professionals who use or deliver CTPs in the three countries in which our partners are based (Brazil, Colombia and South Africa). The aim of this qualitative research is to elicit information about:

(i) Young people’s experiences and meaning of poverty and mental health in these diverse cultures and settings;
(ii) Personal experiences of being involved in the CTP,
(iii) Implementation barriers and facilitators of current programmes; and
(iv) Ideas for future combined CTP and mental health interventions.

The interviews will allow us to gain an in-depth understanding of young people’s experiences with CTPs, how they experience mental health and poverty more generally, and how they view their future, whereas in focus groups we seek to get an understanding of the family and community context in which programmes operate. We will elicit views from young people, families and professionals about the local context and about how programmes operate, the role of programme features and how programmes can be improved to better support young people.

We plan to conduct between 15 and 20 interviews and 3 and 4 focus groups in each of the 3 countries. Sampling and recruitment strategies for interviews and focus group will be tailored to the country setting. Partners will build rapport with the community and potential participants beforehand, through community visits and meetings utilising relationships with community organisations, youth groups and non-government organisations.

For the interviews, we will invite young people who are past or current users of CTPs, and who received or applied for the cash transfer themselves or who received this via their parents. For the focus groups, we will recruit parents who receive cash transfers, practitioners involved in the delivery of programmes and youth leaders from youth organisations.

Focus groups and interviews will be conducted in the local language, audio-recorded, transcribed and translated into English. First, country-specific analysis will be conducted using the complete data (in the mother language where possible). Next, data (in English) will be harmonised across countries by identifying commonalities. We will apply a constant comparative approach towards the coding [48, 52], thus allowing for repeated explorations and reflections with colleagues across countries. Data will be analysed primarily using inductive methods in the form of a framework approach to thematic analysis.

Details on how data for interviews and focus groups will be gathered and analysed following COREQ recommended standards are presented in online resource 1 [53].

### Engaging stakeholders and young people (Objective 5)

A key element of CHANCES-6 is to work in partnership with policy makers and influencers, and other national or local stakeholders. This includes various representatives in governmental and non-governmental organisations (NGOs) who have responsibilities for health and welfare funding, planning and delivery. We will organise high-level policy workshops in each of the countries where our partners are based (i.e., Brazil, Colombia and South Africa), one in the first and one in the last year of the project. In the first round of stakeholder workshops, we will raise awareness of the research aims, seek feedback on methods and approach, and understand interests and capacities in utilising and implementing findings from the research. In addition, we will gather information that will help us understand implementation barriers and facilitators of current programmes, and opportunities for combined provision of CTPs and mental health programmes. The main aim of the second stakeholder workshop will be to discuss implications of the CHANCES-6 findings for policies and programme development, implementation and evaluation. We will plan activities to stay engaged with stakeholders between workshops and identify new stakeholders throughout the project. Partners in each of the countries will facilitate an ongoing dialogue with stakeholders, so we can incorporate their feedback as the research develops based, for example, on policy changes (including in relation to COVID-19), and in the interpretation and presentation of findings.

Additionally, we will develop and use various (social) media channels, outputs and tools to create opportunities for engaging with stakeholders and influence policies in all six countries of the project and beyond. Information and updates on the project, including research findings as they become available during the project, will be provided on the project website (https://www.lse.ac.uk/cpec/chances-6). We will also engage with representatives from international development agencies such as the World Bank, World Health Organisation and UNICEF and with relevant international communities.

Our ultimate beneficiaries are young people living in poverty. Overall, we plan to work with young people throughout the project, by engaging with youth leaders and representatives of organisations which advocate for the rights of young people, locally or nationally. The approach towards engaging with youth will be context-specific, and build on existing partnerships. Young people will be invited to become involved in telling their stories through social media, and by participating in a short films. Young people will become involved in the project, for example in the roles of advisors, advocates, and research staff. This will include involving students from universities, and involving youth in the interpretation of findings of the research.

## Discussion

CHANCES-6 is a multi-disciplinary, multi-site study, which spans six Latin American and African countries. Innovative data collection and analyses—combined with stakeholder engagement—will generate critical knowledge to inform policies and programme designs that consider young people’s mental health when seeking to optimise investment into CTPs. The ultimate aim of this project is to understand how to break the cycle of poverty and mental illness during adolescence to improve young people’s future life chances.

Investments into mental health—both in total as well as in proportion to total health expenditure—are very small in LMICs and treatment is available to very few people. CTPs on the other hand are available and reach a substantial proportion of the population. In the global mental health field, a considerable amount of research effort has gone into designing and evaluating treatment interventions in the past 10–15 years, whereas much less research has been concerned with addressing the social determinants of mental ill health [33]. CHANCES-6 is an opportunity to understand how widely implemented interventions such as cash transfer programmes influence mental health and to develop a more integrated approach to addressing mental health and its social determinants.

There is growing interest among international development agencies and governments in LMICs in understanding the role of poor mental health in maintaining poverty cycles [54]. In this context, CHANCES-6 is particularly salient, for a number of reasons. Firstly, we will generate knowledge on the role of mental health as a mediator and moderator for future life chances of young people living in poverty, and on the causal relationships between poverty and mental health in this population. This addresses an important gap in the evidence as most studies in this area have been descriptive and cross-sectional [40]. Secondly, we will contribute to the emerging evidence on the impact of CTPs on young people’s mental health, and the features of programmes that influence mental health. Thirdly, we will generate knowledge on the role of mental health interventions or support in augmenting the impact of CTPs on life chances. Findings from the economic analyses will provide policy stakeholders with the knowledge of the value of CTPs, and whether including access to mental health support as part of such programmes is likely to increase their economic value. Fourthly, we will generate knowledge about the feasibility, opportunities and barriers in relation to how programmes might be best delivered to improve young peoples’ mental health and life chances. Finally, CHANCES-6 will generate methodological advances for pooling data from diverse longitudinal data sources to estimate mental health and socio-economic effects that are locally relevant and internationally comparable.

## Supplementary Information

Below is the link to the electronic supplementary material.

Supplementary file1 (DOCX 39 KB)

Supplementary file2 (DOCX 27 KB)

## Data Availability

The data sources for the quantitative analysis are secondary sources, which are already publicly available. For the qualitative data analysis, data are not fully shareable as some of the will contain identifiable information. However, findings from the analysis will be published together with details on the research methods and tools.
