# Supplementary material for: Examining the dynamics between young people’s mental health, poverty and life chances in six low- and middle-income countries: protocol for the CHANCES-6 study
Source: Soc Psychiatry Psychiatr Epidemiol. 2021 Jul 19;56(9):1687–703. doi: 10.1007/s00127-021-02043-7 (PMC8286885; doi:10.1007/s00127-021-02043-7)
Supplement: Supplementary file 1 — Supplementary file1 (DOCX 39 KB) [file 127_2021_2043_MOESM1_ESM.docx]

**Study title: Examining the dynamics between young people’s mental health, poverty and life chances in six low- and middle-income countries: protocol for the CHANCES-6 study**

**Journal: Social Psychiatry and Psychiatric Epidemiology**

Annette Bauer ^1^, Ricardo Araya Baltra ^2^, Mauricio Avendano Pabon ^3,4^ , Yadira Díaz ^5^, Emily Garman ^6^, Philipp Hessel ^5^, Crick Lund ^2,6^, Paulo Malvasi ^7^, Alicia Matijasevich ^8^, David McDaid ^1^, A-La Park ^1^, Cristiane Silvestre de Paula ^9^, Annie Zimmerman ^2^, Sara Evans-Lacko ^1^

**Affiliations of the authors**

1 London School of Economics and Political Science, Care Policy and Evaluation Centre, Department of Health Policy, London, United Kingdom

2 King's College London, Health Service & Population Research, Institute of Psychiatry, Psychology and Neuroscience, King’s Global Health Institute, London, United Kingdom

3 King’s College London, Global Health & Social Medicine, London, United Kingdom

4 Harvard School of Public Health, Department of Social and Behavioural Sciences, Boston, United States

5 Universidad de Los Andes, Escuela de Gobierno Alberto Lleras Camargo, Bogotá, Colombia

6 University of Cape Town, Department of Psychiatry and Mental Health, Alan J Flisher Centre for Public Mental Health, Cape Town, South Africa

7 Universidade de São Paulo, Faculdade de Ciências Médicas da Santa Casa de São Paulo, Department of Public Health, São Paulo, Brazil

8 Universidade de São Paulo, Faculdade de Medicina FMUSP, Departamento de Medicina Preventiva, São Paulo, Brazil

9 Universidade Presbiteriana Mackenzie, Programa de Pós-graduação em Distúrbios do Desenvolvimento, São Paulo, Brazil

Email of corresponding author: s.evans-lacko@lse.ac.uk

**Online resource 1**

**Table with details on the conduct of interviews and focus groups for CHANCES-6 study according to COREQ recommendations**

|  | Brazil | Colombia | South Africa |
| --- | --- | --- | --- |
| 1. **INTERVIEWS** | | | |
| **Domain 1: Research team and reflexivity** | | | |
| **Personal characteristics** | | | |
| 1. Interviewer/ facilitator who will conduct the interviews | Paulo Malvasi | Philipp Hessel (PH), Shari Ortiz (SO) | Kauthar Parker (KP), Phatiswa Mhlebi (PM) |
| 2. Credentials of researchers | PhD | PH (PhD, MA, MSc), SO (BA) | KP (BSc Psych Hons), PM (Secondary Education Matriculation) |
| 3. Occupation of researchers | Assistant Professor | PH (Associate Professor), SA (Research Assistant) | KP (research assistant), PM (fieldworker) |
| 4. Gender of researcher | Male | PH (male), SO (female) | KP (female), PM (female) |
| 5. Experience and training of researchers | Anthropologist with twenty years’ experience in qualitative research. Qualitative research training during master's and doctoral degree. Experience in ethnographic research and the use of other qualitative research techniques such as focus groups and in-depth interviews. Conducted qualitative research among youth and poverty in Brazil. | PH: Formal and applied training in qualitative research methods during MA in Sociology and MSc in Social Research Methods. Conducted and directed qualitative research on social pensions and health of older individuals in Colombia.  SO: Formal and applied training in qualitative methods as part of undergraduate program in government and public affairs. | KP: Formal training in qualitative research methods during BSc Hons in Psychology; 11 years of research experience in both qualitative (interviews and focus groups).  PM: 10 years of research experience in qualitative data collection and analysis. Training received at the start of each research project.  KP and PM: formal and applied training in qualitative methods as part of CHANCES-6. |
| **Relationship with participants** | | | |
| 6. Relationship established prior to study commencement | Yes | Yes | No |
| 7. Participant knowledge of researcher (knowledge that participant is provided with about the researcher e.g. personal goals, reasons for doing research) | The recruitment of participants and organization of the research will take place based on a long-term relationship between the researcher and a network of social workers on the outskirts of São Paulo throughout local health services. All participants are provided with participant information sheets and informed consent forms. | Potential participants receive an introduction to the research and researcher from a young person who is part of a network of youth leaders who recruit them into the study. All participants are provided with participant information sheets and informed consent forms. | KP worked as a research assistant in the same non-government organisation which participants are recruited from; potential participants receive an introduction to the research and researcher by staff from non-government organisation. All participants are provided with participant information sheets and informed consent forms. |
| 8. Interviewer characteristics reported (e.g. bias, assumptions, interest in topic) | Researcher interested in topics related to youth, social inequality, mental health and life chances. | PH: Interested in the social determinants of health and particularly the relationship between social protection systems and mental health.  SO: Interested in youth mental health and qualitative methods. | Interviewers interested in topics related to youth and mental health; interviewers are from the same community in which participants are recruited. |
| **Domain 2: Study design** | | | |
| **Theoretical framework** | | | |
| 9. Methodological orientation and Theory | Thematic analysis | Thematic analysis | Thematic analysis |
| **Participant selection** | | | |
| 10. Sampling | Purposive, snowball | Purposive, snowball | Purposive, snowball |
| 11. Method of approach | Face-to-face, phone if face-to-face not feasible (during the COVID-19 pandemic) | Face-to-face, phone if face-to-face not feasible (during the COVID-19 pandemic) | Face-to-face |
| 12. Sample size | 12 | 25 | 16 (8 among Afrikaans-speaking participants, 8 among isiXhosa-speaking participants) |
| 13. Non-participation (i.e. people refused to participate or dropped out) | Not yet known | Not yet known | Not yet known |
| **Setting** | | | |
| 14. Setting of data collection | In community support social services located in two neighborhoods on the outskirts of São Paulo. Via phone (during the COVID-19 pandemic). | In a rented communal space in the neighbourhood where interviewees live. Via phone (during the pandemic). | In a private room provided by the NGO from which participants were recruited. Via phone (during the pandemic). |
| 15. Presence of non-participants | No | No | No |
| 16. Description of sample | Youth aged 18-24, outskirts urban residents, recipients (or former recipients) of cash transfer programme | Youth aged 16-20, rural and urban residents, recipients (or former recipients) of cash transfer programme | Youth aged 15-24; residents in two townships in/near Cape Town (Gugulethu & Khayelitsha), recipients or former recipients of cash transfer programme |
| **Data collection** | | | |
| 17. Interview guide | Yes | Yes | Yes |
| 18. Repeat interviews carried out (including number) | Not yet known | Not yet known | Not yet known |
| 19. Audio/ visual recording | Audio | Audio | Audio |
| 20. Field notes during or after interview | Yes | Yes | Yes |
| 21. Duration | Up to 1 hour | Up to 1 hour | Up to 1 hour |
| 22. Data saturation considered | Additional interviews might be conducted if unresolved issues identified | Additional interviews might be conducted if unresolved issues identified | Additional interviews might be conducted if unresolved issues identified |
| 23. Transcripts returned to participants | No | No | No |
| **Domain 3: Analysis and findings** | | | |
| **Data analysis** | | | |
| 24. Number of data coders | 1 | 2 | 2 |
| 25. Description of the coding tree provided | Country specific coding framework and cross-country coding framework | Planned (e.g. additional focus groups will be conducted if unresolved issues identified) | Planned (e.g. additional focus groups will be conducted if unresolved issues identified) |
| 26. Derivation of themes in advance or derived from the data | Derived from data | Derived from data | Derived from data |
| 27. Software used to manage data | NVivo | NVivo | NVivo |
| 28. Participants provide feedback on findings | Planned (via youth advisory group) | Planned (via youth advisory group) | Planned (via youth advisory group) |
| **Reporting** | | | |
| 29. Quotations presented to illustrate themes and findings | Planned | Planned | Planned |
| 30. Consistency between data and findings | Planned | Planned | Planned |
| 31. Clear presentation of major themes | Planned | Planned | Planned |
| 32. Description of diverse cases and discussion of minor themes | Planned | Planned | Planned |
| 1. **FOCUS GROUPS** | | | |
| **Domain 1: Research team and reflexivity** | | | |
| **Personal characteristics** | | | |
| 1. Interviewer/ facilitator who will conduct the focus groups | Paulo Malvasi | Philipp Hessel (PH), Shari Ortiz (SO) | Kauthar Parker (KP), Phatiswa Mhlebi (PM) |
| 2. Credentials of researchers | PhD | PH (PhD, MA, MSc), SO (BA) | KP (BSc Psych Hons), PM (Secondary Education Matriculation) |
| 3. Occupation of researchers | Assistant Professor | PH (Associate Professor), SA (Research Assistant) | KP (research assistant), PM (fieldworker) |
| 4. Gender of researcher | Male | PH (male), SO (female) | KP (female), PM (female) |
| 5. Experience and training of researchers | Anthropologist with twenty years’ experience in qualitative research. Qualitative research training during master's and doctoral degree. Experience in ethnographic research and the use of other qualitative research techniques such as focus groups and in-depth interviews. Conducted qualitative research among youth and poverty in Brazil. | PH: Formal and applied training in qualitative research methods during MA in Sociology and MSc in Social Research Methods. Conducted and directed qualitative research on social pensions and health of older individuals in Colombia.  SO: Formal and applied training in qualitative methods as part of undergraduate program in government and public affairs. | KP: Formal training in qualitative research methods during BSc Hons in Psychology; 11 years of research experience in both qualitative (interviews and focus groups).  PM: 10 years of research experience in qualitative data collection and analysis. Training received at the start of each research project.  KP and PM: formal and applied training in qualitative methods as part of CHANCES-6. |
| **Relationship with participants** | | | |
| 6. Relationship established prior to study commencement | Yes | Yes | No |
| 7. Participant knowledge of researcher (knowledge that participant is provided with about the researcher) | The recruitment of participants and organization of the research took place based on a long-term relationship between the researcher and a network of social workers on the outskirts of São Paulo. | Participants will receive information via participant information sheet and informed consent form, as well as in presentation at beginning of focus group. | Participants will receive information via participant information sheet and informed consent form. |
| 8. Interviewer characteristics (e.g. bias, assumptions, interest in topic) | Researcher interested in topics related to youth, social inequality, mental health and life chances. |  | Interviewers interested in topics related to youth and mental health; interviewers are from the same community in which participants are recruited |
| **Domain 2: study design** | | | |
| **Theoretical framework** | | | |
| 9. Methodological orientation and Theory | Thematic analysis | Thematic analysis | Thematic analysis |
| **Participant selection** | | | |
| 10. Sampling | Purposive, snowball | Purposive, snowball | Purposive |
| 11. Method of approach | Face-to-face | Face-to-face | Face-to-face |
| 12. Sample size | 4 focus groups (8-12 individuals each one) | 4 focus groups (8-12 individuals per group) | 4 focus groups (6-8 participants per group) |
| 13. Non-participation | Not yet known | Not yet known | Not yet known |
| **Setting** | | | |
| 14. Setting of data collection | In community support centre (social care services) located in participants’ neighborhoods on the outskirts of São Paulo. | In rented communal space located in participants’ neighbourhood. | In facilities of non-government organisation located in participants’ neighbourhood. |
| 15. Presence of non-participants | No | No | No |
| 16. Description of sample | Two focus groups with caregivers and two focus groups with stakeholders, including, social workers and community leaders, residents in the outskirts of São Paulo. | Youth aged 16-20, rural and urban residents, living in lower-income neighbourhoods | 2 focus groups with parents receiving cash transfer programmes; parents Afrikaans/isiXhosa speaking; 2 focus groups with professionals at non-government organisation working with youth; English speaking  Location: in townships near Cape Town (Gugulethu and Khayelitsha) |
| **Data collection** |  |  |  |
| 17. Interview/ topic guide | Yes | Yes | Yes |
| 18. Repeat interviews carried out (including number) | Not yet known | Not yet known | Not yet known |
| 19. Audio/ visual recording | Audio | Audio | Audio |
| 20. Field notes during or after interview | Yes | Yes | Yes |
| 21. Duration | 1-2 hours | 1-2 hours | 1-2 hours |
| 22. Data saturation considered | Additional focus groups might be conducted if unresolved issues identified | Additional focus groups might be conducted if unresolved issues identified | Additional focus groups might be conducted if unresolved issues identified |
| 23. Transcripts returned to participants | Yes | Yes | No |
| **Domain 3: analysis and findings** | | | |
| **Data analysis** | | | |
| 24. Number of data coders | 1 | 2 | 2 |
| 25. Description of the coding tree provided | Country specific coding framework and cross-country coding framework | Country specific coding framework and cross-country coding framework | Country specific coding framework and cross-country coding framework |
| 26. Derivation of themes in advance or derived from the data | Derived from data | Derived from data | Derived from data |
| 27. Software used to manage data | NVivo | NVivo | NVivo |
| 28. Participants provide feedback on findings | No | No | No |
| **Reporting** | | | |
| 29. Quotations presented to illustrate themes and findings | Planned | Planned | Planned |
| 30. Consistency between data and findings | Planned | Planned | Planned |
| 31. Clear presentation of major themes | Planned | Planned | Planned |
| 32. Description of diverse cases and discussion of minor themes | Planned | Planned | Planned |
