# Supplementary material for: Examining the dynamics between young people’s mental health, poverty and life chances in six low- and middle-income countries: protocol for the CHANCES-6 study
Source: Soc Psychiatry Psychiatr Epidemiol. 2021 Jul 19;56(9):1687–703. doi: 10.1007/s00127-021-02043-7 (PMC8286885; doi:10.1007/s00127-021-02043-7)
Supplement: Supplementary file 2 — Supplementary file2 (DOCX 27 KB) [file 127_2021_2043_MOESM2_ESM.docx]

**Study title: Examining the dynamics between young people’s mental health, poverty and life chances in six low- and middle-income countries: protocol for the CHANCES-6 study**

**Journal: Social Psychiatry and Psychiatric Epidemiology**

Annette Bauer ^1^, Ricardo Araya Baltra ^2^, Mauricio Avendano Pabon ^3,4^ , Yadira Díaz ^5^, Emily Garman ^6^, Philipp Hessel ^5^, Crick Lund ^2,6^, Paulo Malvasi ^7^, Alicia Matijasevich ^8^, David McDaid ^1^, A-La Park ^1^, Cristiane Silvestre de Paula ^9^, Annie Zimmerman ^2^, Sara Evans-Lacko ^1^

**Affiliations of the authors**

1 London School of Economics and Political Science, Care Policy and Evaluation Centre, Department of Health Policy, London, United Kingdom

2 King's College London, Health Service & Population Research, Institute of Psychiatry, Psychology and Neuroscience, King’s Global Health Institute, London, United Kingdom

3 King’s College London, Global Health & Social Medicine, London, United Kingdom

4 Harvard School of Public Health, Department of Social and Behavioural Sciences, Boston, United States

5 Universidad de Los Andes, Escuela de Gobierno Alberto Lleras Camargo, Bogotá, Colombia

6 University of Cape Town, Department of Psychiatry and Mental Health, Alan J Flisher Centre for Public Mental Health, Cape Town, South Africa

7 Universidade de São Paulo, Faculdade de Ciências Médicas da Santa Casa de São Paulo, Department of Public Health, São Paulo, Brazil

8 Universidade de São Paulo, Faculdade de Medicina FMUSP, Departamento de Medicina Preventiva, São Paulo, Brazil

9 Universidade Presbiteriana Mackenzie, Programa de Pós-graduação em Distúrbios do Desenvolvimento, São Paulo, Brazil

Email of corresponding author: s.evans-lacko@lse.ac.uk

**Online resource 2:** Table with example of data harmonisation for selected mental health and poverty indicators for Colombia, Mexico and South Africa

|  | COLOMBIA | MEXICO | SOUTH AFRICA |
| --- | --- | --- | --- |
| **Mental health indicators** | | | |
| Mental health problems | Strengths and Difficulties Questionnaire (SDQ);  Including: sleep; good attention span; restless or overactive; sadness; unhappy; tearful; worried; fearful or scared; one good friend | 21 questions about mental health  Including appetite; sleep; sadness; fearful; feeling pessimistic, that things go wrong; difficulty focusing/ reduced productivity; lost interest in things; lonely | Centre for Epidemiological Studies Depression Scale (CES-D) cut off 12  Including: sleep; trouble focusing; depressed; fearful; problems to get going; feeling everything is an effort; lonely |
| Life satisfaction | Feelings regarding 11 aspects of life; degree of satisfaction on 1 to 4 scale | 3 questions about whether life improved in past year and expectations for future 3 years (for community and country) | Current life satisfaction (‘How do you feel about your life as a whole right now?’) on a scale 1 to 10 |
| **Poverty indicators** | | | |
| Household education | Educational achievement   - Average educational level for people aged 15 or older living in household   Literacy   - Percentage of people living in a household who are aged 15 or older and know how to read and write | | |
| Household employment | - Percentage of household members of working age who are not in long-term unemployment (more than 12 months) - Percentage of household members of working age who are employed and affiliated with a pension fund | | |
| Children’ schooling and work | - Percentage of children between the ages of 6 and 16 in the household that attend school - Percentage of children and youths (7–17 years old) within the household that are not suffering from   school lag (according to the national norm)   - Percentage of children between 5 and 17 years in household not working | | |
| Household deprivation | Access to public utilities   - Urban households: no connection to public water systems; rural households: water used for the preparation of food obtained from wells, rainwater, spring sources, water tanks, water carriers or other sources - Urban households: no public sewer system; rural households: toilet without a sewer connection, latrine or no sewage system   Housing conditions   - Urban household: exterior walls built of untreated wood, boards, planks, vegetation (e.g. guadua), zinc, cloth, cardboard, waste material or no exterior walls; rural household: exterior walls built of vegetation (e.g. guadua) zinc, cloth, cardboard, waste materials or no exterior walls - Number of people sleeping per room, excluding kitchen, bathroom and garage | | |
| Health insurance | Percentage of household members over aged 5 or older who are insured by national security and health system | | |
